# Do Canine Behavioural Assessments and Characteristics Predict the Human-Dog Interaction When Walking on a Leash in a Shelter Setting?

**DOI:** 10.3390/ani11010026

**Published:** 2020-12-25

**Authors:** Hao-Yu Shih, Mandy B. A. Paterson, Fillipe Georgiou, Clive J. C. Phillips

**Affiliations:** 1 Centre for Animal Welfare and Ethics, School of Veterinary Science, University of Queensland, Gatton, QLD 4343, Australia; mpaterson@rspcaqld.org.au; 2 Royal Society for the Prevention of Cruelty to Animals Queensland, Brisbane, QLD 4076, Australia; 3 School of Mathematical and Physical Sciences, University of Newcastle, Callaghan, NSW 2308, Australia; fillipegeorgiou@hotmail.com; 4 Curtin University Sustainability Policy (CUSP) Institute, Curtin University, Perth, WA6845, Australia; Clive.Phillips@curtin.edu.au

**Keywords:** on-leash walk, canine behavioural assessment, leash tension, behaviour, verbal cue, body gesture, human-dog interaction, shelter

## Abstract

**Simple Summary:**

We explored 370 human-dog interactions in an animal shelter when volunteers walked shelter dogs on a leash, considering the effects of canine demographics and the results of the shelter’s canine behavioural assessments. Results showed that dogs that were more relaxed during the shelter assessment (i.e., when socialising with humans or being left alone in a new environment) were less reactive on the leash, with lower tension and pulling frequency. Moreover, socialised and relaxed dogs displayed more positive body language, such as tail in a high position, gazing at the handler, and exploring the environment. When walking with these dogs, volunteers utilised fewer verbal guidance cues and body language during the walk. In addition to the canine behaviour assessment, there were correlations between canine demographics (i.e., age, skull shape, body size, and previous ownership history) and the behavioural interaction and humans’ perception. Finally, volunteers perceived the walk as less satisfactory when they needed to pull the leash harder during the walk. This research suggests that the RSPCA behavioural assessment may be useful in predicting the behaviour of shelter dogs when walked by volunteers.

**Abstract:**

Inappropriate leash reactivity is one of the most common problems in shelter dogs, which negatively affects the health of dogs and reduces their adoptability. We explored 370 human-dog interactions, involving 74 volunteers and 111 dogs, in an animal shelter when volunteers walked shelter dogs on a leash, considering the effects of canine demographics and the results of the shelter’s canine behavioural assessments. The interaction was video recorded and coded using ethograms, and a leash tension meter was used to measure the pull strength of dogs and handlers. Results showed that dogs that were more relaxed during the shelter assessment (i.e., when socialising with humans or being left alone in a new environment) were less reactive on the leash, with lower tension and pulling frequency. Moreover, socialised and relaxed dogs displayed more positive body language, such as tail in a high position, gazing at the handler, and exploring the environment. When walking with these dogs, volunteers utilised fewer verbal cues and body language during the walk. In addition to the canine behaviour assessment, there were correlations between canine demographics and the behavioural interaction and humans’ perception. Finally, volunteers perceived the walk as less satisfactory when they needed to pull the leash harder during the walk. This research suggests that the RSPCA behavioural assessment may be useful in predicting the behaviour of shelter dogs when walked by volunteers.

## 1. Introduction

An animal shelter is a challenging environment for dogs, causing both short-term and long-term stress, with acutely elevated cortisol levels within five days of dogs being transferred into a shelter [1]. Moreover, prolonged confinement leads to more problematic behaviours (e.g., decreased activity or excessive auto-grooming [2]), which compromises the animal’s welfare and negatively influences their adoptability [3]. Therefore, positive human-dog interactions that help alleviate the stress of dogs are important in an animal shelter [4]. There are a variety of human-dog interactions that occur routinely in shelters, including petting [4], training [5] and on-leash dog walking [6]. Despite the extended literature on human-dog interactions, limited research has explored the role of human-dog interactions in the shelter context. 

Physical characteristics of dogs influence the human-dog interaction. Compared to adult dogs, puppies are more likely to evoke our nurturing instinct and more quickly and easily form a stronger attachment with humans [7,8]. Smaller dogs are generally perceived as less obedient, more anxious, fearful, excitable and aggressive [9], while larger dogs are thought to be more cooperative and playful [10]. Dogs with wider heads are more likely to display self-grooming but less likely to chase [11]. Nevertheless, small-sized dogs are generally preferred by the general public [3], and there is a trend of people favouring breeds with shorter and wider heads [12,13,14]. 

To evaluate the behaviour of incoming dogs and their adoptability [15,16], many animal shelters have implemented canine behavioural assessments (e.g., SAFER^®^ [16] and Assess-A-Pet™ [17]). In the Royal Society for the Prevention of Cruelty to Animals Queensland (RSPCA Qld), a similar test is conducted on adult dogs around day 5 after arrival to the shelter [18,19]. The canine behavioural assessments measure how dogs react to different stimuli in different situations [15,20]. Controversies have arisen about whether the assessments conducted in animal shelters do indeed predict future household behaviours after adoption [21,22,23]. One argument is that certain behaviours, such as aggression [24] and separation anxiety [25], are context- and stimulus-specific. Since the behavioural assessment is usually done by an examiner who may be perceived as unfamiliar and intimidating to the dog, the result may not be transferable to a situation where an owner interacts with the dog [23,24]. Although such arguments have been raised, behavioural assessments still reliably describe animals’ behaviours within the shelter [19,26]. For example, fear, anxiety and arousal-related behaviours found in the assessment were also observed when dogs were in the shelter kennels [19].

Behaviours of dogs are also related to how we perceive and interact with them. For instance, owners of aggressive English cocker spaniels were reported to be shy, undisciplined, less emotionally stable and tense [27]. Owners are also less likely to engage in shared activities and training with dogs if their dogs are disobedient, aggressive or bark excessively [28]. When it comes to shelters, a less timid dog prefers to play with humans rather than engaging in independent play [29], and dogs spending more time in front of the kennel, laying proximal to the adopter and reacting to humans’ play behaviours attract more preference from potential adopters [30,31]. Problematic behaviours, such as vocalising, house soiling and aggression, negatively affect the owner–dog attachment and significantly increase the risk of unsuccessful dog ownerships, such as owners surrendering dogs to shelters or dogs being returned after adoption [32,33,34]. 

In many dog shelters, on-leash walking is an important part of the human-dog interactions that improves the health of shelter dogs, reduces their in-kennel stress level and facilitates their socialisation [35,36]. However, leash reactivity is one of the most common problems in shelter dogs [37]. Dogs often lunge forwards during on-leash walks, which may damage the soft tissue around the neck and trachea [38] and contribute to increased intraocular pressure [39]. In addition, leash reactivity is reported to be a common problem after adoption [37] and is related to the failure of dog ownerships [40].

An equine rein tension meter measures the force exerted on the rein, which enables researchers and trainers to monitor how a rider communicates with the horse [41,42]. A similar concept is adopted in this study using a custom-made leash tension meter to capture the leash tension when walking a dog on a leash. Moreover, this canine leash tension meter includes an accelerometer in the device which differentiates between human and dog pulling during the walk [43].

Apart from the leash tension meter, behavioural observation using video recording was also used [30,44]. For dogs, the position of the tail, facial expression and ongoing behaviours were recorded, which are directly related to the animal’s welfare [45,46]. In humans, body gestures and verbal cues were measured as they influence the response of the dogs [47,48]. It was hypothesised that dog tolerance to physical contact would be related to higher leash tension, while dogs that were more relaxed in an unfamiliar environment would have lower leash tension [19]. We also hypothesised that dogs would show more friendly signs and have a lower leash tension if the dogs were more accustomed to human interaction. Finally, we hypothesised that handlers would use fewer verbal and gestural cues when dogs were less easily aroused as scored in the behavioural assessment [19] and were older, presumably because they were generally calmer and more obedient [28]. Additionally, handler satisfaction would be negatively associated with leash tension [28].

This article describes the relationship between the results of canine behavioural assessment, canine demographics and human-dog interactions when the dog is walked on a leash, with the focus mainly on canine behavioural assessment. Therefore, results regarding the behavioural assessment will be presented in the main body of the manuscript, followed by relevant demographics. More details on demographic characteristics are presented in the appendices.

This article is a part of a larger research project that explores the behavioural interaction between shelter dogs and volunteers during walks. In this paper, emphasis will be placed on how canine demographics and behavioural assessment results influenced the behavioural interaction while shelter dogs were being walked on leash by volunteers. The effect of human gender has been reported [49] and the effects of body size, body weight, age and the behavioural level of dogs on the leash tension, and the relationship between canine sex and behaviour and leash tension were also reported [43,49]. The influence of other human demographics and personality will be reported in a future publication.

## 2. Materials and Methods 

This study was approved by the Human Research Ethics and Animal Ethics Committees (approval numbers: 2018001570 and SVS/400/18, respectively) of the University of Queensland. 

### 2.1. Study Site

The research was conducted at the Royal Society for the Prevention of Cruelty to Animals, Queensland (RSPCA, Qld) shelter. The housing schedule and shelter environment have been fully described in a previous paper [49]. This study focused on the human-dog interaction during the walks as described in Shih et al. [49].

### 2.2. Subjects

This study investigated 370 walks (370 unique dog-walker pairs), involving 111 shelter dogs and 74 volunteer walkers, with each walker walking 5 different dogs. The classification of the dog behavioural level was based on their performance during the daily walk. Level 1 dogs walked on a loose leash most of the time. Level 2 dogs pulled the leash occasionally. Level 3 dogs tended to pull the leash fiercely. Level 3+ dogs had severe behavioural issues (e.g., aggressiveness or fearfulness) and they might or might not pull the leash harder than level 3 dogs. More details about the behavioural level are described in Shih et al. [49].

### 2.3. RSPCA Canine Behavioural Assessment

Each dog entering RSPCA Qld is behaviourally assessed within 5 days of entry using a standardised assessment. The assessment used is fully described in Clay et al. [19] and comprises the following subtests: Socialisation, Tolerance, Toys, Run and Freeze, Resource Guarding, Toddler Doll, Time Alone and Dog to Dog. In the Socialisation subtest, a dog was allowed to freely explore the room and interact with the handler. Its behaviour and body tension were evaluated. In the Tolerance subtest, the dog’s behaviour, body position and tension were assessed when it was stroked and touched on the feet by the handler. In the Toys subtest, the dog was allowed to play with the toys by itself and with the handler; its behaviour during the interaction was recorded. In the Run and Freeze subtest, the handler ran around in the room and suddenly froze in the corner. The response of the dog was recorded. In the Resource Guarding subtest, the dog was given wet food, dry food and a real bone, consecutively. The examiner assessed its behaviour, body position and tension when the handler was trying to remove the food with a fake hand. In the Toddler Doll subtest, the dog was presented with a fake toddler which mimicked the physical appearance and behaviour of a real child, and its reaction to the toddler was assessed. In the Time Alone subtest, the dog was left alone in the room without any room light (but some natural light through a window) while being videoed. Its behaviour was later evaluated by reviewing the digital recording. Finally, in the Dog to Dog test, the assessed dog was introduced to another dog, and the behaviour of the assessed dog was recorded. This research focused on the human-dog dyad and thus the Dog to Dog subtest was not discussed here.

### 2.4. Canine Leash Tension Meter

The customised canine leash tension (sampling rate: 10 Hz; measuring range: 0–100 kg-force; resolution: 100 g-force) meter was commissioned for this project (RobacScience Australia). The device measures the force exerted on the leash and detects the direction of the pulling (handler versus dog). [43]. 

### 2.5. Measurement of the Dog’s Body

Body size was determined using the body height (cm), body length (cm), body weight (kg) and body condition score (BCS). Body height and length were measured from the ground to the dorsal scapular rim and from the cranial aspect of the shoulder joint to the caudal aspect of the sciatic tuberosity, respectively, using a tape measure [50]. Body weight was obtained from the RSPCA database. The BCS was determined according to the 9-point scale BCS system [51]. Cephalic index (CI) was calculated as the ratio of skull width to skull length. The skull length was measured from the occipital crest to the tip of the nose, and the width was the widest part of the dog’s head (distance between two zygomatic arches) [11].

### 2.6. Study Design

The study design is explained in Shih et al. [49]. Participants were instructed to walk the dog with one hand and only to touch the leash when the dog got tangled. This represented the most common practice in the real world, even though the official advice is to use two hands [52]. After each walk, participants completed a questionnaire about their perspective of the walk (Table 1). 

### 2.7. Data Analysis

#### 2.7.1. Video Recordings of Dog and Human Behaviours

To record the interaction during the walk, a camera (GoPro Hero 7 Silver, GoPro^®^, San Mateo, CA, USA) was mounted on the walker’s head, and, at the same time, the researcher recorded the walk from 10 m behind with an iPhone 7 (Apple Inc., Cupertino, CA, USA). Videos were coded in their entirety by the researcher, who is a veterinarian and a certified dog trainer, with Boris^©^ behaviour observation software [53], using the same method described in Shih et al. [49]. To blind the coder, video coding was completed prior to any analysis of human and canine characteristics. Ten randomly selected videos were recoded to check intra-observer reliability (Cohen’s Kappa = 0.76). Canine behaviours, human verbal cues and human body language were coded using ethograms developed based on previous research [4,45,54,55,56,57], as previously described [49] and modified during practice sessions (Table 2, Table 3 and Table 4). These tables are reproduced from Shih et al. [49] to help with the understanding of this paper.

#### 2.7.2. Leash Tension Analysis

Leash tension and pulling frequency were analysed with MATLAB^®^ (MATLAB^®^ and Statistics Toolbox Release 2018b, The MathWorks, Inc., Natick, MA, USA) using the same approach as described in Shih et al. [49]. 

Every pulling episode can be divided into three phases. Phase one is the “initiation phase”, when either the dog or the handler initiates the pulling, which is marked by an increase in leash tension and acceleration of the device towards the initiator of the pull. Phase two is the “contest phase”, when the other party counteracts the pulling, which is marked by an acceleration in the opposite direction to the initiator and a sharp increase in the leash tension. Finally, phase three is the “losing or winning phase”, when either the party that initiated the pulling wins the contest or the opposite party wins (the party that initiated the pulling loses) the contest. This phase is characterised by a decrease in the leash tension; there should be acceleration towards the “winner”. The leash tension then either returns to the baseline or a new pulling episode characterised by a change in the gradient of the leash tension occurs.

Net maximal tension (NT_max_), maximal tension by dog (DT_max_) and handler (HT_max_) represent the maximal tension throughout the walk, recorded for the dog and handler, respectively. Net mean tension (NT_mean_), mean tension by dog (DT_mean_) and mean tension by handler (HT_mean_) represent the mean tension throughout the walk, recorded for the dog and handler, respectively. Dog pulling frequency (DPF) and handler pulling frequency (HPF) were the frequency of pulling initiated by the dog and handler, respectively.

### 2.8. Statistical Analysis

Statistical analysis was conducted using RStudio Version 1.2.1335 [58] with packages leaps [59], MASS [60], car [61], carData [62], Matrix [63], polycor [64], plyr [65], psych [66], ggpubr [67] and nlme [68]. 

The canine behavioural assessment was transferred into the scoring system based on the effect of each behavioural presentation on the human-dog interaction and safety (Table S1). A higher score in the subtest indicates that dogs displayed more behaviours that would favour the human-dog interaction and safety. One dog had two behavioural assessment results because the shelter staff wanted to reconfirm or monitor the result. For that dog, an average score was calculated. 

This study used the same statistical analysis methods as described in [49]. Bivariate generalised linear models were used for the analysis of each combination of dependent (leash tension, pulling frequency, behaviour and the score of the exit questionnaire) and independent (human and dog demographics, human personality, canine behavioural assessment) variables, followed by generalised linear mixed models for multivariate analyses and repetitions of dogs and walkers. According to the results of bivariate generalised linear models, independent variables with *p* values less than 0.2 and those logically expected to affect the dependent variable, regardless of the *p* value, were included in the generalised linear mixed model. Dependent variables were manually transformed for statistical analysis to meet the assumptions of generalised linear mixed models, including the normality of residual and random effects and homogeneity of variance of residual [49,69].

## 3. Results

### 3.1. Demographics

This study involved 111 shelter dogs, with 58 (52.3%) females and 53 (47.7%) males and all were gonadectomised. The mean age of dogs was 3.74 (± 2.45) years (44.82 ± 29.37 months) old. The mean body height and length were 52.04 (± 6.29) cm and 56.05 (± 5.93) cm, respectively. The mean body weight was 24.87 (± 6.65) kg and the mean body condition score was 4.59 (± 1.07). Finally, the mean cephalic index was 0.58 (± 0.058).

There were 43 (38.74%) stray dogs, 31 (27.93%) surrendered to the RSPCA by owners, 19 (17.12%) returned to the RSPCA by previous adopters and 18 (16.22%) having other or unknown sources. 

### 3.2. Canine Behavioural Assessment, Demographics and Leash Tension/Frequency

The median score and interquartile range (IQR) of each behavioural assessment subtest was as follows: socialisation (median = 3.00, IQR= 1.00), tolerance (median = 4.00, IQR = 3.00), toy (median = 1.00, IQR = 3.00), run and freeze (median = 3.50, IQR = 1.00), resource guarding (median = −3.00, IQR = 1.00), toddler (median = 4.00, IQR = 1.00) and time alone (median = −1.00, IQR = 1.00).

Dogs that were more socialised when exploring the room and interacting with humans were correlated with lower maximal net leash tension (*p* = 0.026). However, dogs that were more tolerant of the human’s physical contact were associated with higher maximal net leash tension (*p* = 0.048). Dogs that were more engaged in playing with toys by themselves or with humans had less pulling frequency (*p* = 0.043). Dogs being more friendly to a model toddler were related to higher maximal net leash tension (*p* = 0.018) and higher pulling frequency created by dogs (*p* = 0.016) and humans (*p* = 0.0003). Dogs exhibiting more reactions (lower score) to time spent alone were correlated with higher mean net leash tension (*p* = 0.018), higher mean (*p* = 0.039) leash tension and pulling frequency (*p* = 0.038) created by dogs and higher maximal (*p* = 0.026) and mean (0.025) leash tension created by humans (Table 5).

Compared to dogs classified as strays, those surrendered by their owners had lower net maximal (*p* = 0.0084) and mean (*p* = 0.0008) leash tension, maximal (*p* = 0.022) and mean (*p* = 0.0003) leash tension created by dogs and maximal (*p* = 0.041) and mean (*p* = 0.0029) leash tension created by humans; dogs returned after a failed adoption were associated with lower maximal leash tension (*p* = 0.047) and pulling frequency by humans (*p* = 0.013) (Table S2).

### 3.3. Canine Behavioural Assessment, Demographics and Canine Behaviour

Dogs that were more socialised when exploring the room and interacting with humans spent a higher percentage of time tracking (*p* = 0.0085) and keeping their tails in a high position (*p* = 0.049) and gazed at handlers more frequently (*p* = 0.036). Dogs that were calmer when seeing a person running and suddenly freezing less frequently displayed gazing (*p* = 0.038) and lip-licking (0.0013) behaviours, but they spent a greater percentage of time sniffing (*p* = 0.013). For dogs showing less resource guarding potential, gazing (*p* = 0.0006) and lip-licking (*p* = 0.0001) were less commonly observed, but these dogs eliminated more often (*p* = 0.036). Finally, dogs being calmer and friendlier toward the fake toddler gazed at handlers less frequently (*p* = 0.045) (Table 6).

The age of the dog was negatively correlated with the percentage of time dogs spent tracking (*p* = 0.012) and the frequency of shaking behaviour (*p* = 0.0021). The cephalic index of the dog was positively related to the percentage of time dogs spent tracking (*p* = 0.0025) but negatively associated with the percentage of time dogs spent panting (*p* = 0.0009). Compared to level 3 dogs, level 2 dogs wagged their tails more often (*p* = 0.038); level 3+ dogs displayed higher percentages of tracking (*p* = 0.0015) and sniffing behaviours (*p* = 0.039) but a lower percentage of panting behaviours (*p* = 0.027) (Table S3). 

### 3.4. Canine Behavioural Assessment, Demographics and Human Behaviour

Less frequent communication (*p* = 0.029), commands (*p* = 0.0079) and giving food treats (*p* = 0.034) were observed when handlers walked dogs that were calmer when seeing a person running and suddenly freezing. When walking dogs with less resource guarding potential, handlers were less likely to communicate (*p* = 0.0025) with them and displayed less body language (*p* = 0.0017) and were less likely to give food treats (*p* = 0.0002) and initiate physical contact (*p* = 0.0008) (Table 7 and Table 8).

The age of the dog was negatively correlated with the frequency of human communication (*p* = 0.043). Compared to level 3 dogs, handlers used more negative verbal cues when walking level 2 dogs (*p* = 0.042). Compared to stray dogs, handlers were more likely to talk to surrendered dogs using a high-pitched voice (*p* = 0.028). The size of the dog was positively related to the frequency of communication (*p* = 0.011), total body language (*p* = 0.02) and physical contact initiated by the handler (*p* = 0.0058) (Tables S4 and S5).

### 3.5. Canine Behavioural Assessment, Demographics and Walking Experience

No significant relationship was observed between the canine behavioural assessment and the walking experience (*p* > 0.05) (Table 9). The age of the dog was positively associated with the score of factor H (*p* = 0.041) and factor D (*p* = 0.0096). Compared to level 3 dogs, level 3+ dogs were correlated with lower scores of factor H (*p* = 0.016) and factor D (*p* = 0.026). Mean leash tension created by humans was negatively related to the factor H score (*p* = 0.0062) (Table S6). 

## 4. Discussion

### 4.1. Canine Behavioural Assessment, Demographics and Leash Tension/Frequency

Dogs being more engaged in playing with toys by themselves or with humans and releasing the toy on command or when traded with treats were less likely to pull on the leash. This finding may indicate that these dogs were more relaxed when interacting with humans and they were more likely to understand and obey human signals when walking on the leash. Dogs that were more friendly and relaxed when facing the fake toddler had higher maximal net leash tension. In addition, these dogs pulled on the leash more frequently and so did the handlers. Since dogs did not encounter any other human being, except the handler, during the walk, it was unlikely that these dogs were attracted by a person or a toddler. Moreover, a previous study on the canine behavioural assessment using a fake toddler or fake cat has shown that instead of testing the dog’s response to a real toddler or a cat, the test is likely testing its response to a foreign object [70,71,72]. Therefore, in our result, it was more likely that these dogs pulled more frequently on the leash because they were more interested in exploring objects around them and handlers were simply responding to the higher pulling frequency by pulling on the leash more frequently too. 

Finally, dogs that were more anxious and reactive when left alone in an unfamiliar environment pulled harder and more frequently, which supports our hypothesis about the negative correlation between the relaxation of dogs in a new environment and leash tension. It might also suggest that these dogs were anxious and attempted to escape from the environment. A better image quality is needed to allow a better examination of the dogs and therefore permit a better understanding of the underlying emotion or motivation of the dog. For handlers, during such interactions, handlers also pulled harder on the leash. This subtest was intended to identify the dog’s potential to develop separation-related issues. Nevertheless, it has been shown that such tests fail to reliably predict the future development of separation-related behaviours after adoption [23]. It might be, however, that, in the subtest, dogs were demonstrating behaviours responding to the foreign environment, which explains why dogs showing more anxious and stress-related signs in the subtest were less relaxed during the walk, even though handlers were around them.

Compared to dogs found as strays, those relinquished by owners had lower leash tension and those returned by adopters had lower pulling frequency created by handlers. This result satisfies the hypothesis that owner relinquished and returned dogs are more familiar with human interaction and thus are more manageable on the leash. However, this result differs from a previous study showing that the prevalence of unruly behaviour (e.g., jumping up, pulling the lead, poor command responding, lack of concentration) between stray and relinquished dogs was not significantly different [73]. 

Dogs that were more socialised when exploring the room and interacting with humans had lower maximal net leash tension, potentially because these dogs were generally more relaxed. In line with our hypothesis, dogs that were more tolerant of human physical contact were associated with higher maximal net leash tension, probably because they tended to ignore the pressure exerted on their bodies when pulling on the leash or were simply less concerned about the human and just eager to go for their walk. However, these results should be interpreted with caution because only net tension was measured.

### 4.2. Canine Behavioural Assessment, Demographics and Canine Behaviour

Dogs that were more socialised when exploring the room and interacting with humans spent a higher percentage of time tracking and keeping their tails high and they more frequently gazed at the handler. Such results satisfy the hypothesis regarding the positive association between canine socialisation and positive body language of dogs. Dogs that explored the exam room were also more likely to explore in other environments, supporting the finding that the RSPCA Qld socialisation test predicts their friendless and sociability in a new environment after adoption [23]. The high tail position and frequent gazing behaviour show that socialised dogs were more confident and more engaged in their interactions with handlers [74,75]. Dogs that were calmer and more relaxed when seeing a person running and freezing less frequently gazed at the handler and displayed lip-licking behaviours, but they spent a higher percentage of time sniffing. Since human-directed gazing and lip-licking can also be interpreted as signs of anxiety or anticipation [76,77], it was possible that these dogs were generally less stressed or aroused when walking on a leash and preferred spending more time exploring the environment through sniffing [76,77]. The sniffing behaviour may also be a displacement signal, indicating conflicting emotions during environmental exploration and mild stress resulting from the novel environment or interacting with the handler [78]. Dogs that were more relaxed in the resource guarding subtest less frequently licked their lips and gazed at the handler but eliminated more frequently. Dogs that were calmer and friendlier toward the fake toddler also gazed at handlers less often. These results might indicate that dogs that were more relaxed when approached during eating and when encountering a foreign object were less defensive and stressed during the walk [76,77]. However, again, results should be interpreted with care as this result might only represent dogs’ in-shelter behaviours [19] because shelter assessment of resource guarding has been shown to unreliably predict their behaviours outside of the shelter post-adoption [79].

The age of the dog was negatively correlated with the time spent tracking during the walk, which supports the previous assumption that older dogs accumulate more experiences and thus they naturally become less engaged in their surroundings, showing less interest in exploration and a reduction in excitement [80]. Older dogs also shook their bodies less frequently when on leash. Body shaking is a recognised displacement behaviour linked to stressful situations involving anxiety and excitement, which is believed to be an attempt to relieve the accumulated stress [81]. In addition, older dogs were found to pull less frequently on the leash [43]; therefore, it might be concluded that compared to younger dogs, older dogs were generally calmer and less stressed and excited, showing less interest in the environment during the walk.

Dogs with a wider head (higher cephalic index) spent more time tracking but less time panting, which seems to contradict the fact that brachycephalic breeds are more likely to pant due to brachycephalic obstructive airway syndrome [82]. The result should be interpreted cautiously because most of the dogs in this study were mesocephalic [83]. Therefore, it is more reasonable to conclude that within mesocephalic breeds, wider-headed dogs may spend more time tracking but less time panting when walking on the leash. The cephalic index of dogs is fully explained by neither the breed groups nor the genetic clusters [84]. Consequently, the finding is less likely due to the breed effect but more likely due to the structural differences, which requires further investigation. 

Compared to level 3 dogs, level 2 dogs wagged their tails more often, which was more commonly seen when interacting with handlers or waiting for handlers to open the gate to enter the next walking section. This may indicate that level 2 dogs enjoyed being around humans more, which is supported by the lower leash tension created by level 2 dogs [43]. Level 3+ dogs showed more tracking and sniffing but less panting behaviours, potentially because they were actively searching for stimuli.

### 4.3. Canine Behavioural Assessment, Demographics and Human Behaviour

Less frequent communication, commands and giving food treats were observed when handlers walked dogs that were calmer upon seeing a person running and freezing. Similarly, handlers were less likely to communicate and displayed less body language, including giving food treats and initiating physical contact when walking dogs that were less defensive and anxious in the resource guarding test. These findings support the hypothesis that handlers would use fewer verbal and gestural cues if dogs were less aroused in the shelter assessment. A possible explanation may be that fewer verbal and physical guides are needed during the walk, when dogs are calm and relaxed, and also such results support previous findings that behaviours associated with fear, anxiety and arousal in the RSPCA Qld assessment predict dogs’ in-shelter behavioural presentations [19].

Our hypothesis that fewer human verbal or physical signals would be observed when walking older dogs was supported. Handlers less frequently used communication and negative verbal cues when interacting with older dogs, showing that older dogs were generally more stable [85], which aligns with the lower pulling frequency observed in older dogs [43]. Handlers were more likely to use negative verbal cues when talking to level 2 dogs compared to level 3 dogs. A possible explanation may be that level 2 dogs were more often involved in interactions with humans and thus were more likely to get excited and needed to be stopped by handlers. Voice pitch was a key factor modulating the behaviour of younger dogs, and humans often communicate with them using a high-pitched voice [86]. In this study, dog age was not found to influence the tendency of humans to use a high-pitched voice for communication. One possible explanation is that most of the dogs in our study were older than 6 months of age. Therefore, these dogs were less likely to be viewed as puppies. Handlers verbally communicated with larger dogs and initiated physical contact more frequently, which may be because handlers could more easily interact and have physical contact with larger dogs due to the shorter physical distance between them. 

### 4.4. Canine Demographics and Walking Experience

Handlers were less satisfied with the interaction and dogs were perceived as less obedient when dogs were younger [87,88], supporting the hypothesis that higher satisfaction would be found when the walk involved an older dog. The adolescent-phase disobedient behaviour has been reported in younger dogs, which corresponds with the peak age at which dogs are relinquished to shelters [88,89]. Interestingly, the reduced obedience of dogs observed in the adolescent phase is only found with respect to the carer who has developed attachment with the dog [88]. The development of disinhibited attachment has been reported in shelter dogs, which characterises quickly forming bonds to new humans after short interactions [90,91]. Future study is encouraged to explore the relationship between adolescent-phase disobedient behaviour and human-dog attachment in the animal shelter setting. 

Compared to level 3 dogs, handlers reported a lower level of satisfaction and rated the dogs as less obedient and supportive when walking level 3+ dogs. However, there was no difference between level 3 and level 2 and 1 dogs, and no significant correlation was observed between the canine behavioural assessment and walking experience. In this study, volunteers were matched with dogs based on training experience and the behaviour of the dog due to safety and welfare concerns, and thus the exit questionnaire might fail to reflect the differences. In addition, the level of each dog was rated by RSPCA Qld staff during the daily walk, which included both walk inside and outside of the research area. To standardise the research, in this study, volunteers were asked to answer the exit questionnaire based on their experiences inside the research area. Differences between level 3 and level 2 and 1 dogs might be more detectable when considering the behavioural presentation of the dog outside of the research area, where more stimuli (e.g., other dogs and humans) are present. Another possibility may be that volunteers were generally more tolerant or preferred not to reveal their true thoughts in the questionnaire despite being told that the survey would be de-identified. In spite of this, the satisfaction score was negatively correlated with the mean leash tension created by the handler, supporting the hypothesis that handlers would be less satisfied with the interaction when they needed to pull the leash harder during the walk. 

### 4.5. Limitations

In this study, there were difficulties in accurately identifying the underlying emotions and motivation of the dog. Similar to the cortisol level and heart rate variability, our leash tension meter may only be able to differentiate the relaxed and aroused states of the dog but cannot specify whether the arousal is due to excitement, anxiety or fear [81,92]. To precisely interpret the underlying emotion and motivation of the dog, a better video quality that records the face and the entire body of the dog, environment and the context of the interaction is needed. Other limitations for this study are described in [49].

## 5. Conclusions

This research explored human-dog interactions with two features. Different from many human-dog interaction studies with participants being owners and dogs being pets [28,56], this study was conducted in a shelter setting, where volunteers generally shared a short-term relationship and a weaker bond with shelter dogs. Additionally, since on-leash walking is an important activity for dogs in a shelter and postadoption, the other feature of this study is that volunteers interacted with dogs when on a leash. 

RSPCA canine behavioural assessment may be useful in predicting the behaviour of shelter dogs when walking on-leash with volunteers. Dogs that were more relaxed during the assessment (e.g., when socialising with humans or being left alone in a new environment) were less reactive on the leash, with lower tension and pulling frequency. Additionally, socialised and relaxed dogs displayed more positive body languages, such as tail in a high position, gazing and exploring the environment, and humans showed fewer verbal cues and body languages during the walk with these dogs. 

In addition, we found correlations between canine demographics and the behavioural interaction and human perception. Demographics included age, skull shape, body size and previous ownership history. Finally, the tension of the leash was related to human perception, with the walk perceived as less satisfactory when volunteers needed to pull the leash harder during the walk. This study may help to enhance volunteers’ experiences when walking shelter dogs on a leash and improve canine welfare in shelters. Matching shelter dogs with potential adopters, considering the demographics of dogs and humans and canine behaviour to achieve a higher rate of successful adoption, may be another possibility [43,49,93,94,95].

## Figures and Tables

**Table 1:** Exit questionnaire used for volunteer walkers (reprinted from Shih et al. [49] for convenience).

1. The dog’s behaviour was good.
2. I could not handle the dog well.
3. I felt comfortable when interacting with the dog.
4. I was physically tense.
5. Overall, this was a good experience.
6. The interaction was challenging for me.
7. The dog did not understand me well.
8. I did not feel that I was helping the dog.
9. I felt supported by the dog.
10. I did not enjoy its company.
11. I would love to walk this dog again on another day.
12. I don’t think this dog is suitable for a non-experienced adopter.
13. I think the dog is ready for adoption.

Each description was rated on a 5-point Likert scale from 1 (strongly disagree) to 5 (strongly agree). Reverse scores were used for negative wording for the calculation of mean scores. Human satisfaction factor (Factor H) utilised responses to questions 2, 3, 4, 5, 6, 10, 11. A higher score of factor H represented a higher satisfaction of the walk. Walker’s perception of dog factor (Factor D) utilised responses to questions 1, 7, 8, 9, 12, 13. A higher score of factor D indicated that the dog was considered more supportive and better behaved. Details about the statistical justification of the questionnaire are described in [49].

**Table 2:** Ethogram of canine behaviour [49].

Behaviour	Description	Behaviour Type	Reference
Track	Dog uses nose to follow a scent along the ground.	State event	[54]
Sniff	Dog explores or expresses stress or appeasement by orientating its nose to an object, wall or ground, and the dog stands still.	State event	[54]
Eliminate-mark	Dog defecates or urinates in sitting, squatting or standing positions.	Point event	[45]
Shake	Dog shakes its body or head.	Point event	
Pant	Dog breathes vigorously with its mouth wide open.	State event	[54]
Gaze	Dog looks toward the handler.	Point event	[54]
Lip-lick	Dog shows its tongue and moves its tongue along the upper lip or snout.	Point event	[54]
Tail wag	Dog moves its tail from side to side.	State event	[4]
Tail high	Dog holds its tail upright.	State event	[55]

Point event: the number of times the event was observed. State event: the duration of the observed event.

**Table 3:** Ethogram of human verbal cues [49].

Behaviour	Description	Behaviour Type	Reference
Sit	Volunteer asks the dog to sit.	Point event	
Command	Volunteer talks to the dog with an utterance containing a single command, exclusive of the “sit” command (e.g., “Stay!” “Come!” “Let’s go!” ).	Point event	[56]
Attention seeking	Volunteer tries to get the attention of the dog by calling the name of the dog and/or using the utterance of “Look!” and/or clicking the tongue.	Point event	[56]
High-pitched voice	Volunteer talks to the dog using a high-pitched voice or baby-talk expressions.	Point event	[4]
Praise	Volunteer talks to the dog with a positive utterance (e.g., “Great!” “Well done!” “Good dog!”).	Point event	[4,56]
Negative verbal cue	Volunteer talks to the dog with a negative utterance (e.g., “No!” “Bad dog!” “Don’t …” “Stop …” “Let the lead (it) go”).	Point event	
Communication	Volunteer communicates with the dog by asking the dog some questions. (e.g., “Which way do you want to go?” “What are you sniffing at?” “Do you want to fetch?” “Do you want to drink?”)	Point event	[57]

Point event: the number of times the event was observed. State event: the duration of the observed event.

**Table 4:** Ethogram of human body language [49].

Behaviour	Description	Behaviour Type	Reference
Gestural	Volunteer displays voluntary hand movement directed towards the dog (e.g., referential point, patting his/her own thigh, luring the dog with a hand or food).	Point event	[4,56]
Physical contacts	Physical contacts initiated by the volunteer. Including contacts when treats were given.	Point event	
Food reward	Food is given to the dog including directly giving it, tossing it or placing it on the ground or an object.	Point event	

Point event: the number of times the event was observed.

**Table 5:** Generalised linear mixed model of the effect of canine behavioural assessment on leash tension and pulling frequency.

Behavioural Assessment	Log_10_NT_max_	Log_10_NT_mean_	Log_10_DT_max_	Log_10_DT_mean_	Log_10_DPF^1^	Log_10_HT_max_	Log_10_HT_mean_	Log_10_HPF ^1^
Socialisation	*β* −0.071	*β* −0.0038	*β* −0.049	*β* −0.04	*β* 0.078	*β* −0.055	*β* −0.032	*β* 0.022
	SE 0.032	SE 0.024	SE 0.035	SE 0.024	SE 0.055	SE 0.034	SE 0.025	SE 0.05
	*p* 0.026	*p* 0.87	*p* 0.16	*p* 0.092	*p* 0.16	*p* 0.11	*p* 0.19	*p* 0.65
Tolerance	*β* 0.031	*β* 0.011	*β* 0.03	*β* 0.016	*β* 0.029	*β* 0.032	*β* 0.02	*β* 0.0084
	SE 0.015	SE 0.011	SE 0.017	SE 0.011	SE 0.027	SE 0.016	SE 0.012	SE 0.026
	*p* 0.048	*p* 0.32	*p* 0.076	*p* 0.18	*p* 0.29	*p* 0.052	*p* 0.1	*p* 0.74
Toy	*β* 0.022	*β* −0.0059	*β* 0.011	*β* −0.0036	*β* −0.1	*β* 0.011	*β* −0.0035	*β* −0.041
	SE 0.028	SE 0.021	SE 0.031	SE 0.021	SE 0.05	SE 0.03	SE 0.022	SE 0.046
	*p* 0.43	*p* 0.78	*p* 0.72	*p* 0.86	*p* 0.043	*p* 0.72	*p* 0.87	*p* 0.38
Run and Freeze	*β* −0.0055	*β* −0.019	*β* −0.015	*β* −0.0038	*β* −0.068	*β* −0.0095	*β* −0.003	*β* −0.067
	SE 0.025	SE 0.02	SE 0.027	SE 0.019	SE 0.042	SE 0.027	SE 0.019	SE 0.037
	*p* 0.82	*p* 0.34	*p* 0.59	*p* 0.84	*p* 0.11	*p* 0.72	*p* 0.86	*p* 0.075
Resource Guarding	*β* −0.023	*β* −0.01	*β* −0.029	*β* −0.027	*β* 0.044	*β* −0.026	*β* −0.02	*β* 0.059
	SE 0.026	SE 0.019	SE 0.028	SE 0.019	SE 0.045	SE 0.028	SE 0.02	SE 0.041
	*p* 0.37	*p* 0.6	*p* 0.31	*p* 0.17	*p* 0.32	*p* 0.35	*p* 0.32	*p* 0.15
Toddler	*β* 0.055	*β* 0.0081	*β* 0.049	*β* 0.014	*β* 0.098	*β* 0.041	*β* 0.0088	*β* 0.13
	SE 0.023	SE 0.017	SE 0.025	SE 0.017	SE 0.041	SE 0.025	SE 0.018	SE 0.037
	*p* 0.018	*p* 0.64	*p* 0.053	*p* 0.42	*p* 0.016	*p* 0.11	*p* 0.62	*p* 0.0003
Time Alone	*β* −0.11	*β* −0.041	*β* −0.09	*β* −0.073	*β* −0.17	*β* −0.11	*β* −0.081	*β* −0.05
	SE 0.047	SE 0.038	SE 0.051	SE 0.035	SE 0.08	S 0.05	SE 0.036	SE 0.072
	*p* 0.018	*p* 0.28	*p* 0.078	*p* 0.039	*p* 0.038	*p* 0.026	*p* 0.025	*p* 0.49

Tension and pulling frequency were analysed in log_10_ transformation. NT_max_: maximal net leash tension. NT_mean_: mean net leash tension. DT_max_: maximal leash tension caused by dog. DT_mean_: mean leash tension caused by dog. HT_max_: maximal leash tension caused by handler. HT_mean_: mean leash tension caused by handler. DPF: dog pulling frequency. HPF: handler pulling frequency. *β*: regression coefficient. SE: standard error of *β*. *p*: *p* value of the model. ^1^ Pulling frequency = (Number of pulls) / (walking duration). A pull was defined as a bout of force greater than 0.1% of the dog’s body weight force.

**Table 6:** Generalised linear mixed model of the effect of canine behavioural assessment on canine behaviour.

Behavioural Assessment	Track (%)	Tail High (%)	Tail Wag (%)	Gaze (no./sec)	Lip-Lick (no./sec)	Eliminate-Mark (no./sec)	Shake (no./sec)	Pant (%)	Sniff (%)
Socialisation	*β* 0.023	*β* 0.053	*β* 0.015	*β* 0.014	*β* 0.011	*β* 0.0042	*β* 0.0004	*β* 0.0053	*β* −0.0057
	SE 0.0086	SE 0.027	SE 0.013	SE 0.0067	SE 0.0064	SE 0.002	SE 0.00043	SE 0.013	SE 0.0074
	*p* 0.0085	*p* 0.049	*p* 0.27	*p* 0.036	*p* 0.091	*p* 0.059	*p* 0.35	*p* 0.68	*p* 0.44
Tolerance	*β* −0.00097	*β* 0.021	*β* 0.0081	*β* −0.0026	*β* 0.0035	*β* 0.00031	*β* 0.000053	*β* 0.0066	*β* 0.00082
	SE 0.004	SE 0.012	SE 0.0062	SE 0.0033	SE 0.0029	SE 0.0011	SE 0.00019	SE 0.0062	SE 0.0037
	*p* 0.81	*p* 0.09	*p* 0.19	*p* 0.42	*p* 0.24	*p* 0.77	*p* 0.78	*p* 0.29	*p* 0.83
Toy	*β* 0.0025	*β* 0.0045	*β* −0.014	*β* 0.0	*β* −0.0026	*β* −0.0032	*β* −0.00017	*β* 0.011	*β* 0.0065
	SE 0.0075	SE 0.023	SE 0.012	SE 0.006	SE 0.0057	SE 0.0019	SE 0.00036	SE 0.012	SE 0.0066
	*p* 0.74	*p* 0.84	*p* 0.24	*p* 0.099	*p* 0.65	*p* 0.099	*p* 0.64	*p* 0.35	*p* 0.33
Run and Freeze	*β* −0.004	*β* 0.00627	*β* −0.018	*β* −0.01	*β* −0.016	*β* 0.0028	*β* −0.00043	*β* −0.0086	*β* 0.015
	SE 0.0062	SE 0.019	SE 0.01	SE 0.0052	SE 0.0049	SE 0.0015	SE 0.00032	SE 0.009	SE 0.0061
	*p* 0.53	*p* 0.74	*p* 0.08	*p* 0.038	*p* 0.0013	*p* 0.072	*p* 0.19	*p* 0.34	*p* 0.013
Resource Guarding	*β* 0.0034	*β* 0.033	*β* −0.0017	*β* −0.02	*β* −0.022	*β* 0.0036	*β* 0.0002	*β* −0.016	*β* −0.0018
	SE 0.0067	SE 0.021	SE 0.011	SE 0.0056	SE 0.0054	SE 0.0017	SE 0.00035	SE 0.01	SE 0.0068
	*p* 0.61	*p* 0.12	*p* 0.88	*p* 0.0006	*p* 0.0001	*p* 0.036	*p* 0.57	*p* 0.14	*p* 0.79
Toddler	*β* −0.0016	*β* 0.0097	*β* 0.0087	*β* −0.011	*β* −0.0072	*β* 0.0021	*β* −0.00004	*β* −0.0026	*β* −0.0037
	SE 0.0064	SE 0.02	SE 0.011	SE 0.0053	SE 0.0052	SE 0.0016	SE 0.00033	SE 0.0097	SE 0.0066
	*p* 0.8	*p* 0.62	*p* 0.42	*p* 0.045	*p* 0.17	*p* 0.21	*p* 0.9	*p* 0.79	*p* 0.57
Time Alone	*β* −0.0042	*β* −0.015	*β* 0.0059	*β* −0.012	*β* −0.0067	*β* 0.0024	*β* −0.00077	*β* −0.026	*β* 0.0017
	SE 0.013	SE 0.037	SE 0.021	SE 0.011	SE 0.01	SE 0.0033	SE 0.00062	SE 0.02	SE 0.013
	*p* 0.74	*p* 0.69	*p* 0.77	*p* 0.27	*p* 0.5	*p* 0.45	*p* 0.22	*p* 0.19	*p* 0.9

Track (%): tracking time (s)/total walking time (s) × 100%. Tail high (%): tail high time (s)/total walking time (s) × 100%, analysed in power of 7. Tail wag (%): tail wagging time (s)/total walking time (s) × 100%, analysed in power of 0.3. Gaze (no./sec): number of gazes / time when the dog’s head was visible in the Gopro video (s), analysed in power of 0.4. Lip-lick (no./sec): number of lip-licks/time when the dog’s head was visible in the Gopro video (s), analysed in power of 0.4. Eliminate-mark (no./sec): number of eliminate-marks/total walking time (s), analysed in power of 0.6. Shake (no./sec): number of shakes/total walking time (s), analysed in power of 0.8. Pant (%): painting time (s)/time when the dog’s head was visible in the Gopro video (s) × 100%, analysed in power of 0.5. Sniff (%): sniffing time (s)/total walking time (s) × 100%, analysed in power of 0.5.β: regression coefficient. SE: standard error of β. *p*: *p* value of the model.

**Table 7:** Generalised linear mixed model of the effect of canine behavioural assessment on human verbal cue.

Behavioural Assessment	Total Verbal Cue (no./sec) ^1^	Attention Getter (no./sec) ^2^	Communication (no./sec) ^2^	Negative Verbal cue (no./sec) ^2^	Praise (no./sec) ^1^	High-Pitched Voice (no./sec) ^1^	Command (no./sec) ^1^
Socialisation	*β* 0.00049	*β* 0.0033	*β* 0.00021	*β* 0.0007	*β* −0.006	*β* 0.0026	*β* 0.0018
	SE 0.0077	SE 0.0058	SE 0.0038	SE 0.0033	SE 0.0045	SE 0.0042	SE 0.0052
	*p* 0.95	*p* 0.57	*p* 0.96	*p* 0.83	*p* 0.18	*p* 0.54	*p* 0.74
Tolerance	*β* −0.0012	*β* −0.00042	*β* −0.0029	*β* −0.00073	*β* 0.00024	*β* −0.001	*β* −0.00047
	SE 0.0038	SE 0.0029	SE 0.0019	SE 0.0017	SE 0.002	SE 0.0019	SE 0.0024
	*p* 0.76	*p* 0.88	*p* 0.13	*p* 0.66	*p* 0.91	*p* 0.59	*p* 0.85
Toy	*β* −0.002	*β* 0.0051	*β* 0.0019	*β* 0.003	*β* 0.00068	*β* 0.0041	*β* 0.0019
	SE 0.007	SE 0.0054	SE 0.0036	SE 0.003	SE 0.0039	SE 0.0034	SE 0.0044
	*p* 0.78	*p* 0.34	*p* 0.6	*p* 0.33	*p* 0.86	*p* 0.23	*p* 0.67
Run and Freeze	*β* −0.012	*β* −0.0096	*β* −0.0076	*β* −0.003	*β* −0.005	*β* −0.0052	*β* −0.011
	SE 0.0059	SE 0.005	SE 0.0034	SE 0.003	SE 0.0037	SE 0.0034	SE 0.004
	*p* 0.052	*p* 0.057	*p* 0.029	*p* 0.31	*p* 0.18	*p* 0.13	*p* 0.0079
Resource Guarding	*β* −0.013	*β* −0.0024	*β* −0.011	*β* −0.0027	*β* −0.003	*β* −0.0036	*β* −0.0066
	SE 0.0067	SE 0.0051	SE 0.0036	SE 0.0032	SE 0.0038	SE 0.0033	SE 0.0042
	*p* 0.052	*p* 0.64	*p* 0.0025	*p* 0.41	*p* 0.43	*p* 0.28	*p* 0.12
Toddler	*β* −0.0013	*β* 0.0047	*β* −0.0044	*β* −0.0022	*β* 0.0016	*β* 0.0021	*β* −0.0036
	SE 0.0064	SE 0.0053	SE 0.0037	SE 0.0031	SE 0.0038	SE 0.0033	SE 0.0042
	*p* 0.84	*p* 0.38	*p* 0.23	*p* 0.48	*p* 0.68	*p* 0.53	*p* 0.4
Time Alone	*β* 0.023	*β* 0.0169400	*β* 0.0045	*β* −0.0055	*β* 0.012	*β* 0.0034	*β* 0.0089
	SE 0.012	SE 0.01	SE 0.007	SE 0.006	SE 0.007	SE 0.006	SE 0.0077
	*p* 0.056	*p* 0.098	*p* 0.52	*p* 0.37	*p* 0.088	*p* 0.57	*p* 0.25

^1^ Analysed after transformation to the power of 0.5. ^2^ Analysed after transformation to the power of 0.4. β: regression coefficient. SE: standard error of β. *p*: *p* value of the model.

**Table 8:** Generalised linear mixed model of the effect of canine behavioural assessment on human body language.

Behavioural Assessment	Total Body Language (no./sec) ^1^	Food Reward (no./sec)	Hand Gesture (no./sec) ^2^	Physical Contact (no./sec) ^1^
Socialisation	*β* 0.002	*β* 0.000025	*β* 0.0024	*β* 0.00048
	SE 0.0085	SE 0.00026	SE 0.0044	SE 0.0069
	*p* 0.81	*p* 0.92	*p* 0.58	*p* 0.94
Tolerance	*β* −0.0013	*β* 0.000091	*β* −0.000036	*β* −0.0017
	SE 0.0041	SE 0.00012	SE 0.0021	SE 0.0033
	*p* 0.75	*p* 0.46	*p* 0.98	*p* 0.6
Toy	*β* 0.0013	*β* −0.00027	*β* −0.0015	*β* 0.003
	SE 0.0077	SE 0.00023	SE 0.004	SE 0.0062
	*p* 0.87	*p* 0.25	*p* 0.7	*p* 0.63
Run and Freeze	*β* −0.012	*β* −0.00048	*β* −0.0074	*β* −0.0026
	SE 0.0073	SE 0.00022	SE 0.0038	SE 0.0058
	*p* 0.1	*p* 0.034	*p* 0.051	*p* 0.65
Resource Guarding	*β* −0.026	*β* −0.00084	*β* −0.0069	*β* −0.022
	SE 0.008	SE 0.00022	SE 0.0039	SE 0.0064
	*p* 0.0017	*p* 0.0002	*p* 0.078	*p* 0.0008
Toddler	*β* −0.011	*β* −0.00023	*β* −0.0026	*β* −0.0099
	SE 0.0077	SE 0.00024	SE 0.004	SE 0.0061
	*p* 0.16	*p* 0.33	*p* 0.52	*p* 0.11
Time Alone	*β* 0.0074	*β* 0.00053	*β* 0.00056	*β* 0.016
	SE 0.014	SE 0.00043	SE 0.0073	SE 0.011
	*p* 0.61	*p* 0.22	*p* 0.94	*p* 0.17

^1^ Analysed after transformation to the power of 0.5. ^2^ Analysed after transformation to the power of 0.4. β: regression coefficient. SE: standard error of β. *p*: *p* value of the model.

**Table 9:** Generalised linear mixed model of the effect of canine behavioural assessment on volunteers’ walking experience. Factor H represented human satisfaction factor and factor D represented walker’s perception of dog factor.

Behavioural Assessment	Factor H ^1^	Factor D
Socialisation	*β* 281,928	*β* 0.0053
	SE 222,792	SE 0.032
	*p* 0.21	*p* 0.87
Tolerance	*β* −20,161	*β* 0.0057
	SE 103,723	SE 0.016
	*p* 0.85	*p* 0.72
Toy	*β* 218,989	*β* −0.0082
	SE 186,487	SE 0.027
	*p* 0.24	*p* 0.76
Run and Freeze	*β* −185,587	*β* −0.0026
	SE 179,140	SE 0.028
	*p* 0.3	*p* 0.93
Resource Guarding	*β* −144,747	*β* −0.014
	SE 178,912	SE 0.029
	*p* 0.42	*p* 0.64
Toddler	*β* −112,153	*β* 0.02
	SE 183,151	SE 0.029
	*p* 0.54	*p* 0.5
Time Alone	*β* 98,600	*β* 0.014
	SE 357,493	SE 0.052
	*p* 0.78	*p* 0.79

^1^ Analysed after transformation to the power of 10. β: regression coefficient. SE: standard error of β. *p*: *p* value of the model.

## Data Availability

The data presented in this study are available on request from the corresponding author. The data are not publicly available in order to protect participants’ personal privacy.

## Supplementary Materials

The following are available online at https://www.mdpi.com/2076-2615/11/1/26/s1, Table S1: Scoring of canine behavioural assessment. Details of each subtest are described in [19], Table S2: Generalised linear mixed model of the effect of canine demographics on leash tension and pulling frequency, Table S3: Generalised linear mixed model of the effect of canine demographics on canine behaviour, Table S4: Generalised linear mixed model of the effect of canine demographics on human verbal cue, Table S5: Generalised linear mixed model of the effect of canine demographics on human body language, Table S6: Generalised linear mixed model of the effect of canine demographics and leash tension caused by humans on volunteers’ walking experience.
