# Supplementary material for: Do Canine Behavioural Assessments and Characteristics Predict the Human-Dog Interaction When Walking on a Leash in a Shelter Setting?
Source: Animals (Basel). 2020 Dec 25;11(1):26. doi: 10.3390/ani11010026 (PMC7823978; doi:10.3390/ani11010026)
Supplement: Supplementary file 1 [file animals-11-00026-s001.pdf]

animals

MDPI

Article

Do Canine Behavioural Assessments and Characteristics Predict the Human-Dog Interaction When Walking on a Leash in a Shelter Setting?

Hao-Yu Shih, Mandy B.A. Paterson, Fillipe Georgiou and Clive J.C. Phillips

Table 1. Scoring of canine behavioural assessment. Details of each subtest were described in [19].

Response	Score
**Subtest 1: Socialisation**	
Explores the room	+1
Does not socialise with the human	−1
Solicits handler immediately	+1
Comes when called	+1
Does not respond to handler's calls, handler must approach to pick up the leash	0
Stays with handler, with or without physical contact	+1
Moves away from handler	−1
Moves away from handler during attention	−1
Leans into handler's touch during attention	+1
Body is relaxed and neutral during attention	+1
Body is tense during attention	−1
Solicits handler for more following attention directly following attention	+1
Moves away from handler after attention	0
**Subtest 2: Tolerance**	
Procedure is completed while dog is standing	+1
Sits when touched	+1
Lays down and/or begins rolling over to back	+1
Becomes frontal in stance facing the handler	+1
Procedure is unable to be completed due to risk of injury	−1
Procedure is unable to be completed due to the dog struggling to avoid	−1
Body is relaxed	+1
Body is tensed and/or offers a freeze	−1
Tail base is high	+1
Tail base is neutral / medium	0
Tail base is low	−1
Mouth closes when touched	−1
Attempts to move away, end of leash	−1
Air snaps	−1
Mouths with med-hard strength	−1
Bites	−1
**Subtest 3: Toys**	
Engages in play behaviors immediately	+1
Brings tennis ball back to handler	+1
Gives or drops tennis ball on verbal cue	+1
Seeks out the ball and then disinterested	0
Engages in independent play, able to trade for treat	+1
Engages in independent play, unable to trade for treat	0

Becomes intense and aroused in play activity	−1
Unsafe to engage in play, with med to hard mouthiness, air snaps etc.	−1
Guards the toy	−1
Shows no interest	0
**Subtest 4: Run and Freeze**	
Stays near the handler	+1
Moves away from handler	0
Moves away from handler with fearful response	−1
Runs with handler	+1
Body relaxed and balanced	+1
Body is tense	−1
Tail is stiff and high during run	0
Jumps up making no physical contact with handler	−1
Jumps up making gentle contact with handler	−1
Jumps up making hard contact with handler	−1
Snaps toward the handler	−1
Looks to handler with soft eyes	+1
Looks to handler with hard eyes	−1
Looks to handler with dilated eyes	−1
Mounts handler	−1
Mouths with med-hard strength	−1
Mouths with escalating intensity	−1
**Subtest 5: Resource Guarding**	
Shows no interest in food	0
Level 1–2 resource guarding	−1
Level 3–4 resource guarding	−2
**Subtest 6: Toddler Doll**	
Wags tail loosely with a relaxed body carriage	+1
Keeps tail high with relaxed body carriage	+1
Keeps tail low or tucked with body with weight shifted backwards	−1
Keeps tail high with body weight shifted forward	0
Offensive aggression displayed - forward stance, growling, lunging	−1
Defensive aggression displayed - weight shifted backward, vocalization	−1
Increases distance from the doll	−1
Approaches immediately with relaxed body and soft eye contact	+1
Hesitant to approach, avoiding eye contact, body may be tense	−1
Makes nose contact with hand(s), feet, and/or face	+1
Remains with the handler, body relaxed	+1
Decreases distance	0
**Subtest 7: Time Alone**	
No risk for separation anxiety	0
Mild risk for separation anxiety	−1
Moderate risk for separation anxiety	−2
Severe risk for separation anxiety	−3

Level 1–2 resource guarding: dog remains relaxed either disengage from food to interact with the person or does not disengage from food. Level 3–4 resource guarding: dog becomes tense and aware at the human approach. + : the response is beneficial to human-dog interaction, safety and is generally favoured by people. 0 : the response is neither beneficial nor detrimental to human-dog interaction, safety and is neither favoured nor disliked by people. − : the response is detrimental to human-dog interaction, safety and is generally not favoured by people.

Table S2. Generalised linear mixed model of the effect of canine demographics on leash tension and pulling frequency.

Dependent variables / Independent variables		$Log_{10}NT_{max}$	$Log_{10}NT_{mean}$	$Log_{10}DT_{max}$	$Log_{10}DT_{mean}$	DPF^a	$Log_{10}HT_{max}$	$Log_{10}HT_{mean}$	HPF^a
Cephalic index	*β*		0.39			0.48			0.97
	SE	--	0.49	--	--	0.86	--	--	0.78
	p		0.42			0.58			0.22
	**Stray**								
	μ	4.13	0.65	3.54	1.29	0.2	3.49	1.27	0.2
	SD	2.43	0.3	2.1	0.58	0.13	2.25	0.6	0.14
	**Owner surrender**								
	μ	3.23	0.52	2.84	1.01	0.18	2.66	1	0.17
	SD	1.44	0.2	1.47	0.37	0.12	1.25	0.36	0.11
	β	−0.24	−0.22	−0.22	−0.25	−0.074	−0.19	−0.21	−0.17
	SE	0.088	0.063	0.096	0.066	0.15	0.094	0.068	0.14
	p	0.0084	0.0008	0.022	0.0003	0.63	0.041	0.0029	0.23
	**Return**								
Source	μ	3.71	0.56	3.03	1.09	0.15	2.78	1.07	0.14
	SD	2.26	0.21	1.9	0.37	0.12	1.55	0.32	0.1
	β	*β* −0.2	−0.14	−0.19	−0.148	0.032	−0.24	−0.13	−0.43
	SE	0.11	0.082	0.12	0.082	0.19	0.12	0.085	0.17
	P	0.079	0.091	0.11	0.076	0.87	0.047	0.12	0.013
	**Other**								
	μ	3.72	0.63	3.38	1.2	0.23	3.17	1.2	0.22
	SD	1.49	0.23	1.5	0.39	0.16	1.32	0.41	0.12
	β	−0.094	−0.05	−0.033	−0.064	−0.133	0.00038	−0.039	−0.18
	SE	0.11	0.078	0.12	0.08	0.19	0.11	0.083	0.17
	p	0.39	0.52	0.78	0.42	0.47	0.99	0.64	0.27

Tension and pulling frequency were analyzed in log_{10} transformation. Dog age, behavioural level and size (body height, body length, weight and body condition score) were reported in [43], and dog sex was reported in [49]. NT_{max}: maximal net leash tension. NT_{mean}: mean net leash tension. DT_{max}: maximal leash tension caused by dog. DT_{mean}: mean leash tension caused by dog. HT_{max}: maximal leash tension caused by handler. HT_{mean}: mean leash tension caused by handler. DPF: dog pulling frequency. HPF: handler pulling frequency. [a.] Pulling frequency = (Numbers of pulls) / (walking duration). A pull was defined as a bout of force greater than 0.1% of the dog's body weight force. μ: mean (before transformation) (kg force). SD: standard deviation of μ. *β*: regression coefficient. SE: standard error of *β*. *p*: *p* value of the model. --: Not

included in the generalized linear mixed model because the independent variable had high p-values in the bivariate regression model.

Table S3. Generalized linear mixed model of the effect of canine demographics on canine behaviour.

Dependent variables / Independent variables		Track (%)	Tail high (%)	Tail wag (%)	Gaze (no./sec)	Lip-lick (no./sec)	Eliminate-mark (no./sec)	Shake (no./sec)	Pant (%)	Sniff (%)
Age	β	−0.00085	0.000013	−0.00083	−0.000098	−0.00012	0.00012	−0.00005	0.0003	--
	SE	0.00034	0.0011	0.00056	0.00027	0.00027	0.000089	0.000016	0.00052	
	p	0.012	0.99	0.14	0.72	0.66	0.18	0.0021	0.56	
Cephalic index	β	0.3	--	--	−0.11	--	0.02	0.0049	−0.66	--
	SE	0.13			0.11		0.033	0.0068	0.196	
	p	0.025			0.33		0.55	0.47	0.0009	
Walking level	Level 1									
	μ / median	μ 15.87	median 85.34	median 2.03	median 0.01	median 0.01	median 0.01	median <0.01	median 0.17	μ 15.47
	SD / IQR	SD 15.59	IQR 18.82	IQR 4.02	IQR 0.01	IQR 0.01	IQR 0.01	IQR <0.01	IQR 2.86	SD 9.39
	β	0.047	0.023	0.13	0.015	--	--	−0.0028	−0.008	0.06
	SE	0.042	0.12	0.069	0.036			0.0021	0.066	0.044
	p	0.27	0.85	0.071	0.68			0.2	0.9	0.18
	Level 2									
	μ / median	μ 14.22	median 90.49	median 0.09	median 0.01	median 0.01	median <0.01	median <0.01	median 7.19	μ 10.61
	SD / IQR	SD 9.9	IQR 34.82	IQR 2.98	IQR 0.02	IQR 0.02	IQR 0.01	SD <0.01	IQR 13.02	SD 8.78
	β	0.016	0.039	0.061	0.01	--	--	0.00087	0.029	−0.021
	SE	−0.017	0.042	0.029	0.014			0.00089	0.026	0.017
	p	0.35	0.36	0.038	0.46			0.33	0.27	0.21
	Level 3									
	μ / median	μ 15.01	median 92.59	Median <0.01	median 0.01	median <0.01	median 0.01	median <0.01	median 8.49	μ 9.95
	SD / IQR	SD 11.6	IQR 11.49	IQR 1.88	IQR 0.01	IQR 0.01	IQR 0.01	SD <0.01	IQR 13.42	SD 6.74
	Level 3 +									
	μ / median	μ 28.04	median 53.3	Median 3.57	median 0.02	median 0.01	median <0.01	median <0.01	median 3.22	μ 15.34
	SD / IQR	SD 12.22	IQR 76.05	IQR 7.22	IQR 0.02	IQR 0.01	IQR 0.01	IQR <0.01	IQR 5.27	SD 6.25
	β	0.14	−0.11	0.12	0.035	--	--	0.0021	−0.14	0.095
	SE	0.042	0.11	0.071	0.037			0.0022	0.062	0.046
	p	0.0015	0.32	0.093	0.35			0.36	0.027	0.039
Source	Stray									
	μ / median	μ 16.71			median 0.01	median < 0.01	Median <0.01	median <0.01		μ 10.2

	SD / IQR	SD 11.66	median<br>89.31<br>IQR 35.92	median<br>0.73<br>IQR 3.03	IQR 0.01	IQR 0.01	IQR 0.01	IQR <0.01	median<br>8.81<br>IQR 15.24	SD 8.27
	Return<br>μ / median<br>SD / IQR<br>*β*<br>SE<br>*p*	Return<br>μ 14.93<br>SD 8.55<br>0.0039<br>0.03<br>0.9	Return<br>median<br>91.2<br>IQR 20.03<br>--	Return<br>median<br><0.01<br>IQR 2.85<br>--	Return<br>median 0.01<br>IQR 0.01<br>--	Return<br>median <0.01<br>IQR 0.01<br>--	Return<br>median < 0.01<br>IQR < 0.01<br>−0.00059<br>0.0074<br>0.94	Return<br>median <0.01<br>IQR <0.01<br>−0.00039<br>0.0014<br>0.78	Return<br>median<br>2.86<br>IQR 11.03<br>−0.045<br>0.045<br>0.33	Return<br>μ 11.09<br>SD 7.05<br>--
	Owner Surrender<br>μ / median<br>SD / IQR<br>*β*<br>SE<br>*p*	μ 14.1<br>SD 12.57<br>−0.022<br>0.024<br>0.36	median<br>95.54<br>IQR 12.46<br>--	median<br><0.01<br>IQR 2.03<br>--	median 0.01<br>IQR 0.02<br>--	median 0.01<br>IQR 0.02<br>--	median <0.01<br>IQR 0.01<br>0.00019<br>0.0059<br>0.98	median <0.01<br>IQR <0.01<br>−0.000077<br>0.0011<br>0.95	median<br>8.41<br>IQR 12.64<br>0.05<br>0.036<br>0.17	μ 10.23<br>SD 7.07<br>--
	Other<br>μ / median<br>SD / IQR<br>*β*<br>SE<br>*p*	μ 13.42<br>SD 9.76<br>−0.013<br>0.028<br>0.63	median<br>90.33<br>IQR 12.03<br>--	median<br><0.01<br>IQR 1.96<br>--	median 0.01<br>IQR 0.02<br>--	median <0.01<br>IQR 0.01<br>--	median 0.01<br>IQR 0.01<br>−0.0058<br>0.0071<br>0.42	median <0.01<br>IQR <0.01<br>−0.000015<br>0.0013<br>0.99	median<br>6.18<br>IQR 13.36<br>0.025<br>0.042<br>0.55	μ 10.73<br>SD 9.11<br>--
Dog size [1]	*β*<br>SE<br>*p*	--	--	--	--	0.00000005<br>0.00000004<br>0.17	--	−0.000000001<br>0.000000002<br>0.66	0.0000001<br>0.00000007<br>0.14	<0.00000001<br>0.00000005<br>0.99

Dog sex was reported in [49]. Track (%): tracking time (s)/total walking time (s) × 100%. Tail high (%): tail high time (s)/total walking time (s) × 100%, analysed in power of 7. Tail wag (%): tail wagging time (s)/total walking time (s) × 100%, analysed in power of 0.3. Gaze (no./sec): Numbers of gazes / time when the dog's head was visible in the Gopro video (s), analysed in power of 0.4. Lip-lick (no./ses): Numbers of lip-licks/time when the dog's head was visible in the Gopro video (s), analysed in power of 0.4. Eliminate-mark (no./s): Numbers of eliminate-marks/total walking time (s), analysed in power of 0.6. Shake (no./sec): Numbers of shakes/total walking time (s), analysed in power of 0.8. Pant (%): painting time (s)/time when the dog's head was visible in the Gopro video (s) × 100%, analysed in power of 0.5. Sniff (%): sniffing time (s)/total walking time (s) × 100%, analysed in power of 0.5. Dog size is the interaction of the dog's body height, body length, body weight, and body condition score (9-point scale) [51]. μ: mean (before transformation). SD: standard deviation of μ. IQR: interquartile range. *β*: regression coefficient. SE: standard error of *β*. *p*: *p* value of the model. --: Not included in the generalized linear mixed model because the independent variable had high p-values in the bivariate regression model.

Table S4. Generalised linear mixed model of the effect of canine demographics on human verbal cue.

Dependent variables / Independent variables		Total verbal cues (no./sec) [1]	Attention getter (no./sec) [2]	Communication (no./sec) [2]	Negative verbal cue (no./sec) [2]	Praise (no./sec) [1]	High-pitched voice (no./sec) [1]	Command (no./sec) [1]
	β	−0.00046	−0.00037	−0.00034	−0.00018			
Age	SE	0.00032	0.00024	0.00017	0.00014	--	--	--
	p	0.16	0.13	0.043	0.021			
	β		−0.16			0.168	−0.067	
Cephalic index	SE	--	0.13	--	--	0.094	0.072	--
	p		0.19			0.076	0.35	
	Level 1							
	µ / median			median <0.01	median <0.01		median <0.01	
	SD / IQR	µ 0.06	µ 0.01	IQR <0.01	IQR <0.01	µ 0.02	IQR 0.01	µ 0.03
	β	SD 0.04	SD 0.01	−0.0066	0.011	SD 0.02	−0.008	SD 0.02
	SE	--	--	0.022	0.021	--	0.023	--
	p			0.77	0.58		0.73	
	Level 2							
	µ / median			median <0.01	median <0.01		median <0.01	
	SD / IQR	µ 0.09	µ 0.02	IQR 0.01	IQR <0.01	µ 0.02	IQR 0.02	µ 0.03
	β	SD 0.06	SD 0.02	0.016	0.018	SD 0.03	0.0088	SD 0.03
Walking level	SE	--	--	0.011	0.009	--	0.01	---
	p			0.13	0.042		0.38	
	Level 3							
	µ / median	µ 0.08	µ 0.02	median <0.01	median <0.01	µ 0.02	median <0.01	µ 0.03
	SD / IQR	SD 0.07	SD 0.02	IQR <0.01	IQR <0.01	SD 0.02	IQR 0.01	SD 0.03
	Level 3+							
	µ / median			median <0.01	median <0.01		median 0.01	
	SD / IQR	µ 0.11	µ 0.03	IQR 0.01	IQR <0.01	µ 0.02	IQR 0.02	µ 0.05
	β	SD 0.12	SD 0.04	−0.0022	−0.0038	SD 0.02	0.031	SD 0.06
	SE	--	--	0.025	0.022	--	0.024	--
	p			0.93	0.86		0.2	
	Stray							
Source	µ / median	µ 0.09	µ 0.02	median <0.01	median <0.01	µ 0.02	median <0.01	µ 0.03
	SD / IQR	SD 0.07	SD 0.02	IQR 0.01	IQR <0.01	SD 0.03	IQR 0.02	SD 0.04
	Return							

	μ / median	μ 0.08	μ 0.02	median <0.01	median <0.01	μ 0.02	median <0.01	μ 0.03
	SD / IQR	SD 0.05	SD 0.02	IQR 0.01	IQR <0.01	SD 0.02	IQR 0.01	SD 0.03
	β	--	--	--	--	--	−0.008	0.022
	SE						0.014	0.018
	p						0.56	0.23
	Owner surrender							
	μ / median	μ 0.08	μ 0.02	median <0.01	median <0.01	μ 0.02	median <0.01	μ 0.03
	SD / IQR	SD 0.06	SD 0.02	IQR 0.01	IQR <0.01	SD 0.03	IQR 0.01	SD 0.03
	β	--	--	--	--	--	−0.024	−0.01
	SE						0.011	0.014
	p						0.028	0.49
	Other	Other	Other	Other	Other	Other	Other	Other
	μ / median	μ 0.09	μ 0.02	median <0.01	median <0.01	μ 0.03	median <0.01	μ 0.03
	SD / IQR	SD 0.07	SD 0.02	IQR 0.01	IQR <0.01	SD 0.03	IQR 0.02	SD 0.02
	β	--	--	--	--	--	−0.014	0.011
	SE						0.013	0.017
	p						0.29	0.5
Dog size [3]	*β*	--	--	0.0000001	0.00000002	--	--	--
	SE			0.00000002	0.00000002			
	p			0.011	0.42			

Dog sex was reported in [49]. [1] Analysed after transformation to the power of 0.5. [2] Analysed after transformation to the power of 0.4. [3] Dog size is the interaction of the dog's body height, body length, body weight, and body condition score (9-point scale) [51]. μ: mean (before transformation). IQR: interquartile range. SD: standard deviation of μ. *β*: regression coefficient. SE: standard error of *β*. *p*: *p* value of the model. --: Not included in the generalized linear mixed model because the independent variable had high *p*-values in the bivariate regression model.

Table S5. Generalized linear mixed model of the effect of canine demographics on human body language.

Dependent variables / Independent variables		Total body language (no./sec) [1]	Food reward (no./sec)	Hand gesture (no./sec) [2]	Physical contact (no./sec) [1]
Age	*β*	--	--	−0.14	--
	SE			0.096	
	p			0.16	
Cephalic index	*β*	--	0.00078	--	0.036
	SE		0.0056		0.14

	p		0.89		0.8
Walking level	Level 1				
	median	<0.01	<0.01	<0.01	<0.01
	IQR	<0.01	<0.01	<0.01	<0.01
	β	−0.055	--	−0.018	−0.047
	SE	0.052		0.027	0.042
	p	0.29		0.52	0.26
	Level 2				
	median	<0.01	<0.01	<0.01	<0.01
	IQR	0.01	<0.01	<0.01	<0.01
	β	−0.0051	--	0.009	−0.021
	SE	0.02		0.011	0.017
	p	0.81		0.4	0.23
	Level 3				
	median	<0.01	<0.01	<0.01	<0.01
	IQR	0.01	<0.01	<0.01	0.01
	Level 3+				
	median	0.01	<0.01	<0.01	<0.01
	IQR	0.02	<0.01	0.01	0.01
	β	0.022	--	0.017	−0.0031
	SE	0.053		0.029	0.042
	p	0.68		0.56	0.94
Source	Stray				
	median	<0.01	<0.01	<0.01	<0.01
	IQR	0.01	<0.01	<0.01	<0.01
	Return				
	median	0.01	<0.01	<0.01	<0.01
	IQR	<0.01	<0.01	<0.01	<0.01
		--	--	--	--
	Owner sur-				
	render	<0.01	<0.01	<0.01	<0.01
	median	0.01	<0.01	<0.01	0.01
	IQR	--	--	--	--
	Other				

	median	<0.01	<0.01	<0.01	<0.01
	IQR	0.01	<0.01	<0.01	0.01
		--	--	--	--
Dog size [3]	*β*	0.00000012	--	--	0.00000012
	SE	0.00000005			0.00000004
	p	0.02			0.0058

Dog sex was reported in [49]. [1] Analysed after transformation to the power of 0.5. [2] Analysed after transformation to the power of 0.4. [3] Dog size is the interaction of the dog's body height, body length,f body weight, and body condition score (9-point scale) [51]. μ: mean (before transformation). IQR: interquartile range. SD: standard deviation of μ. *β*: regression coefficient. SE: standard error of *β*. *p*: *p* value of the model. --: Not included in the generalized linear mixed model because the independent variable had high *p*-values in the bivariate regression model.

Table 6. Generalised linear mixed model of the effect of canine demographics and leash tension caused by humans on volunteers' walking experience.

Dependent variables / Independent variables		Factor H [1]	Factor D
	β	17148	0.0033
Age	SE	8329	0.0013
	p	0.041	0.0096
	β	−2175064	
Cephalic index	SE	4330148	--
	p	0.62	
	Level 1		
	μ / median	median 5	μ 4.46
	SD / IQR	IQR 0	SD 0.64
	β	634392	−0.099
	SE	1228016	0.21
	p	0.61	0.63
	Level 2		
	μ / median	median 4.86	μ 4.33
	SD / IQR	IQR 0.57	SD 0.65
	β	499090	0.099
Walking level	SE	547599	0.084
	p	0.36	0.24
	Level 3		
	μ / median	median 4.86	μ 4.31
	SD / IQR	IQR 0.43	SD 0.61
	Level 3+		
	μ / median	median 4.14	μ 3.64
	SD / IQR	IQR 0.86	SD 0.87
	β	−3247938	−0.52
	SE	1328344	0.23
	p	0.016	0.026
	Stray		
	μ / median	median 4.86	μ 4.17
	SD / IQR	IQR 0.71	SD 0.71
	Return		
	μ / median	median 4.86	
	SD / IQR	IQR 0.43	μ 4.33
	β	90214	SD 0.73
	SE	759687	--
	p	0.91	
Source	Owner surrender	Owner surrender	
	μ / median	median 5	Owner surrender
	SD / IQR	IQR 0.36	μ 4.4
	β	525956	SD 0.55
	SE	583122	--
	p	0.37	
	Other		
	μ / median	4.86	
	SD / IQR	IQR 0.29	μ 4.34
	β	789479	SD 0.53
	SE	666421	--

	p	0.24	
	β	91669	−0.016
HT_{max}	SE	181225	0.031
	p	0.61	0.6
	β	−1963226	−0.28
HT_{mean}	SE	707425	0.11
	p	0.0062	0.011
	β	439	
Dog size [2]	SE	423	--
	p	0.3	

Dog sex was reported in [49]. [1] Analysed after transformation to the power of 10. [2] Dog size is the interaction of the dog's body height, body length, body weight, and body condition score (9-point scale) [51]. μ: mean (before transformation). IQR: interquartile range. SD: standard deviation of μ. β: regression coefficient. SE: standard error of β. p: p value of the model. --: Not included in the generalized linear mixed model because the independent variable had high p-values in the bivariate regression model.
